# EF-24, a Curcumin Analog, Inhibits Cancer Cell Invasion in Human Nasopharyngeal Carcinoma through Transcriptional Suppression of Matrix Metalloproteinase-9 Gene Expression

**DOI:** 10.3390/cancers15051552

**Published:** 2023-03-01

**Authors:** Shih-Chi Su, Chung-Han Hsin, Yen-Ting Lu, Chun-Yi Chuang, Yu-Ting Ho, Fang-Ling Yeh, Shun-Fa Yang, Chiao-Wen Lin

**Affiliations:** 1 Whole-Genome Research Core Laboratory of Human Diseases, Chang Gung Memorial Hospital, Keelung 204, Taiwan; 2 Department of Dermatology, Drug Hypersensitivity Clinical and Research Center, Chang Gung Memorial Hospital, Linkou 333, Taiwan; 3 Department of Otolaryngology, Chung Shan Medical University Hospital, Taichung 402, Taiwan; 4 School of Medicine, Chung Shan Medical University, Taichung 402, Taiwan; 5 Institute of Medicine, Chung Shan Medical University, Taichung 402, Taiwan; 6 Department of Medical Research, Chung Shan Medical University Hospital, Taichung 402, Taiwan; 7 Department of Biochemistry and Molecular Biology, University of Massachusetts, Amherst, MA 01003, USA; 8 Institute of Oral Sciences, Chung Shan Medical University, Taichung 402, Taiwan; 9 Department of Dentistry, Chung Shan Medical University Hospital, Taichung 402, Taiwan

**Keywords:** nasopharyngeal carcinoma, curcumin analog, cancer metastasis, matrix metalloproteinase-9

## Abstract

**Simple Summary:**

EF-24 has exhibited many anti-cancer properties and enhanced bioavailability over curcumin. We demonstrated that through interfering with the nuclear translocation of NF-κB and the activation of JNK, EF-24 inhibited MMP-9 gene transcription, repressing NPC invasion. These findings provide potential avenues for the use of curcumin analogs with increased bioavailability in managing this devastating disease.

**Abstract:**

Cancer metastasis is a main cause of failure in treating subjects with nasopharyngeal carcinoma (NPC) and is frequently linked to high death rates. EF-24, an analog of curcumin, has exhibited many anti-cancer properties and enhanced bioavailability over curcumin. Nevertheless, the effects of EF-24 on the invasiveness of NPC are poorly understood. In this study, we demonstrated that EF-24 effectively inhibited TPA-induced motility and invasion responses of human NPC cells but elicited very limited cytotoxicity. In addition, the TPA-induced activity and expression of matrix metalloproteinase-9 (MMP-9), a crucial mediator of cancer dissemination, were found to be reduced in EF-24-treated cells. Our reporter assays revealed that such a reduction in MMP-9 expression by EF-24 was transcriptionally mediated by NF-κB via impeding its nuclear translocation. Further chromatin immunoprecipitation assays displayed that the EF-24 treatment decreased the TPA-induced interaction of NF-κB with the MMP-9 promoter in NPC cells. Moreover, EF-24 inhibited the activation of JNK in TPA-treated NPC cells, and the treatment of EF-24 together with a JNK inhibitor showed a synergistic effect on suppressing TPA-induced invasion responses and MMP-9 activities in NPC cells. Taken together, our data demonstrated that EF-24 restrained the invasiveness of NPC cells through the transcriptional suppression of MMP-9 gene expression, implicating the usefulness of curcumin or its analogs in controlling the spread of NPC.

## 1. Introduction

Nasopharyngeal carcinoma (NPC), a neoplasm originating in the part of the throat connecting the back of the nose to the back of the oral cavity, is particularly frequent in East and Southeast Asia [1]. Apart from inherited factors and exposure to the Epstein–Barr virus (EBV), numerous environmental parameters related to unique ethnic practices and lifestyles (e.g., habitual use of tobacco products and preserved food) are known to be involved in the susceptibility to NPC [2]. These predisposing factors over different geographical areas largely elucidate the high heterogeneity in the global prevalence, histologic classifications, and therapy outcomes for this cancer. At present, the main treatment for NPC is radiation therapy alone (for early-stage tumors) or combined chemo-radiotherapy (for advanced diseases). However, nearly 20% of patients receiving primary therapy still developed recurrent tumors or metastatic dissemination [3,4], which are usually resistant to the mainstay of treatment and linked to high death rates [5]. Thus, improving our knowledge of the molecular basis of disseminated NPC is central to the development of new treatment options for ultimately alleviating this aggressive malignancy.

Cancer cell invasion and metastasis are hallmarks of tumors that require malignant cells to move from the original site and propagate at a remote location [6,7]. The metastatic dissemination of NPC comprises various molecular and cellular processes carried out by both malignant and non-malignant cells within the tumor microenvironment [8,9,10,11]. These mechanisms involve the orchestration of signal cascades, the modulation of cell-matrix adhesion, the breakdown of the extracellular matrix (ECM) by matrix metalloproteinases (MMPs), cytoskeletal rearrangement, the induction of cell mobility and angiogenic responses, evading apoptosis, and an epithelial-mesenchymal transition (EMT). TPA (12-O-Tetradecanoylphorbol-13-acetate) is a phorbol ester commonly employed in biomedical studies to promote cancer dissemination, mainly through the activation of protein kinase C (PKC) [12]. The impact of TPA-mediated PKC signaling on cancer metastasis has been well-documented in various cancer types [13,14,15,1613-16].

Curcumin, a polyphenol derived from the rhizome of *Curcuma longa*, exhibits many oncostatic properties in a myriad of tumors [17,18,19]. However, the clinical use of this natural compound is largely restrained by its low solubility and poor bioavailability [20]. To address these issues, efforts toward the development of curcumin derivatives and analogs were made to improve current cancer treatments [21,22,23,24,25,26,27,28]. Among these synthetic analogs of curcumin, EF-24 demonstrated anti-cancer effects and displayed an enhanced bioavailability compared to curcumin [29,30]. Mounting evidence has indicated that EF-24 has impeded cancer progression by employing multiple and interrelated mechanisms, such as the inhibition of NF-κB [31] and MAPK [32,33] signaling, the suppression of hypoxia-inducible factor-1α (HIF-1α) expression [34], glucose metabolism [35], and the regulation of reactive oxygen species (ROS) production [36,37]. However, the impact of EF-24 on NPC metastasis remains unexplored. In the present study, we evaluated the effect of EF-24 on the invasiveness of NPC cells and investigated the molecular mechanisms associated with EF-24-regulated NPC cell migration, aiming to offer potential avenues for the use of curcumin analogs with enhanced bioavailability in combating this devastating malignancy.

## 2. Materials and Methods

### 2.1. NPC Cell Culture and Reagents

HONE-1 cells were obtained from the Food Industry Research and Development Institute (Hsinchu, Taiwan). NPC-39 and NPC-BM, derived from patients with NPC [38], were kind gifts from Dr. MK Chen, Department of Otolaryngology, Changhua Christian Hospital, Changhua, Taiwan. The cell culture was maintained in RPMI-1640 medium at 37 °C in a humidified atmosphere of 5% CO_2_. EF-24 was purchased from Sigma-Aldrich (St. Louis, MO, USA) and prepared in dimethyl sulphoxide (DMSO, Sigma-Aldrich, St. Louis, MO, USA). Where indicated, the cells were pretreated with EF-24 for 1 h and then incubated with 50 ng/mL TPA (Sigma-Aldrich, St. Louis, MO, USA) for 23 h, followed by the subsequent experiments.

### 2.2. Cell Viability Assay

The cytotoxic effects of EF-24 were evaluated by assessing the cell viability using a microculture tetrazolium test (MTT), as previously stated [39]. After the pretreatment of various EF-24 concentrations for 1 h, the cells were incubated with TPA for 23 h, followed by an MTT assay. Cell viability was determined by the generation of formazan, which was measured spectrophotometrically at 563 nm in a spectrophotometer (DU640, Beckman Instruments, Fullerton, CA, USA).

### 2.3. Wound Healing Assay

The cells were cultured in 6 cm plates for 24 h until full confluence. Serum starvation of the cells was performed overnight before making a scratch with a pipette tip [40]. Then, cell debris was washed out, and the cell culture was maintained in a conditioned medium containing the indicated concentrations of EF-24 and TPA. Images of the cell cultures were taken at 0 and 12 h by using an Olympus CKX41 phase-contrast microscope (Olympus Corporation, Tokyo, Japan) at 100× magnification.

### 2.4. Cell Migration and Invasion Assay

Cell migration and invasion were evaluated by a modified Boyden chamber system coated without and with 10 μL of Matrigel (25 mg/50 mL; BD Biosciences), respectively [41,42]. Briefly, the cells were pretreated with the indicated concentrations of EF-24, in the absence or presence of TPA for 24 h, and were subsequently placed at a cell density of 10^4^ cells per well on the membrane filters with a pore size of 8 μm in serum-free media for 24 h. Cell migration or invasion was counted with an Olympus CKX41 microscope (Olympus Corporation, Tokyo, Japan).

### 2.5. Gelatin Zymography

A gelatin zymography protease assay was used to measure the gelatinolytic activities of MMP-9 in the culture medium, as stated previously [43]. In brief, the conditioned medium was subjected to 0.1% gelatin (Sigma-Aldrich, St. Louis, MO, USA) and 8% SDS-PAGE. Followed by the electrophoresis, the gels were washed with 2.5% Triton X-100, soaked with a reaction buffer (10 mM CaCl2, 0.01% NaN3, and 40 mM Tris-HCl, pH 8.0) for 24 h at 37 °C, and were subsequently stained with Coomassie Brilliant Blue R-250 (Sigma-Aldrich, St. Louis, MO, USA).

### 2.6. Immunoblotting and Immunofluorescence

Protein lysates were harvested, subjected to SDS-PAGE, and transferred to Immobilon PVDF membranes (Millipore) [44]. The nuclear protein extraction was performed by using a Nuclear Extraction Kit (ab113474, Abcam) [45]. Antibodies against the following proteins were used for detection: Anti-p38a (612168) from BD Biosciences (Bedford, MA, USA); Anti-NF-κB p65 (51-0500) from Invitrogen (Carlsbad, CA, USA); Anti-Lamin B2 (ab151735) from Abcam (Cambridge, UK); Anti-phospho-p38 mitogen-activated protein kinase (MAPK) (Thr180/Tyr182), Anti-p44/42 MAPK (ERK1/2), Anti-phospho-p44/42 MAPK (ERK1/2), Anti-SAPK/JNK, Anti-phospho-SAPK/JNK (Thr183/Tyr185) antibodies from Cell Signaling Technology (Danvers, MA, USA); and HRP-conjugated secondary antibodies (Dako). Densitometric analyses of immunoblots were conducted by using ImageJ software. For immunofluorescence, the fixation was conducted in 4% paraformaldehyde, and the permeabilization was performed by using PBS containing 0.5% Triton X-100. The cells were then blocked by PBS containing 1% goat serum, 1% BSA, 0.2% sodium azide, and 0.1% Triton X-100. Following hybridization with a polyclonal anti-p-65 antibody (1:100 dilution; 51-0500, Invitrogen) for 2 h and FITC-conjugated goat anti-rabbit IgG (1:50 dilution; Jackson ImmunoResearch) for 1 h, the cells were stained with DAPI and mounted on glass slides with an antifading, aqueous mounting medium (Biomeda, Foster City, CA, USA).

### 2.7. Quantitative PCR

The total RNA was isolated by using an RNeasy Mini Kit (Qiagen), and cDNA was prepared using an AccuScript High-Fidelity 1st Strand cDNA Synthesis Kit (Stratagene). The primers were designed using Beacon Designer software, such that amplicons were 100–200 bp. Real-time PCR was carried out on a Bio-Rad iCycler iQ Multicolor Real-Time PCR Detection system using the iQ SYBR Green Supermix (Bio-Rad, Hercules, CA, USA), as described previously [46]. Primer sequences were as follows: MMP-9: 5′-CAACATCACCTATTGGATCC-3′ (forward) and 5′-CGGGTGTAGAGTCTCTCGCT-3′ (reverse) and GAPDH: GAPDH: 5′-CGGAGTCAACGGATTTGGTCGTAT-3′ (forward), 5’- AGCCTTCTCCATGGTGGTGAAGAC-3′ (reverse). The quantification of relative expression levels was performed based on the standard curves for both MMP-9 and a constitutively expressed gene, GAPDH.

### 2.8. Reporter Assay

A reporter assay was performed by the Luciferase Assay System (Promega, Madison, WI, USA) to analyze the activity of the luciferase. The vector encoding the MMP-9 promoter/reporter gene was a kind gift from Professor JL Ko (Chung Shan Medical University, Taichung, Taiwan). The constructs containing NF-κB-Luc, SP-1-Luc, or AP-1-Luc sequences were obtained from Stratagene (La Jolla, CA, USA). The mutant MMP-9 promoter/reporter vectors were constructed as described previously [47]. Prior to the treatment of EF-24 and TPA, NPC cells were co-transfected with a β-galactosidase expression vector pCH110 (Pharmacia, Piscataway, NJ, USA), pGL-3-basic, and MMP-9 promoter plasmids, by using a Lipofectamine™ 2000 Transfection Reagent (Invitrogen, Carlsbad, CA, USA) for 16 h. The cell lysates were collected, and luciferase activities normalized to a β-galactosidase internal control were assessed with a luciferase assay kit (Sigma).

### 2.9. Chromatin Immunoprecipitation (ChIP) Assay

NPC cells were pretreated with EF-24 for 1 h and were subsequently incubated with TPA for 23 h. A ChIP assay was conducted using the ChIP assay kit (ab500, Abcam) [48]. In brief, the cells were harvested, fixed with 1% paraformaldehyde and quenched with glycine at room temperature. The cells were lysed with the ChIP lysis buffer, and genomic DNA was sheared by sonication to produce DNA fragments of 200–700 bp. The sheared chromatin was immunoprecipitated with antibodies specific to the NF-κB p65 (51-0500, Invitrogen) and protein A Sepharose beads (Invitrogen). The NF-κB-bound chromatin fragments were analyzed using a PCR, using specific primers F-5′-GCCATGTCTGCTGTTTTCTAGAGG-3′ and R-5′-CACACTCCAGGCTCTGTCCTCTTT-3′ for the MMP-9 promotor.

### 2.10. Statistical Analysis

Data represent averages ± standard deviation (SD) of at least three separate experiments. A *p* value of <0.05 was considered statistically significant by using a one-way analysis of variance with Tukey’s post-hoc test.

## 3. Results

### 3.1. EF-24 Restricted Cell Motility and Invasion but Not Cell Viability in TPA-Induced NPC Cells

**Figure 1:**
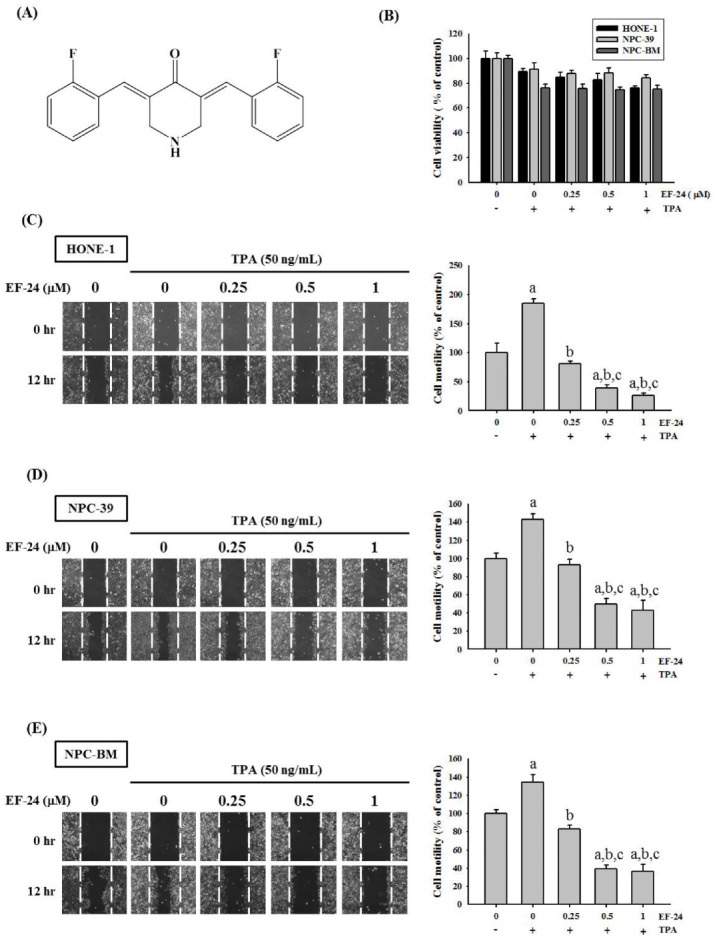
EF-24 inhibited TPA-induced cell motility but did not render cytotoxicity in human NPC cells. (**A**) Structural formula of EF-24. (**B**) Prior to examining for cell viability, NPC cell lines were incubated with different concentrations of EF-24 for 1 h and then treated with TPA for an additional 23 h. Data represented the means ± SD from at least three separate experiments. (**C**–**E**) Wound closure of HONE-1 (**C**), NPC-39 (**D**), or NPC-BM cells (**E**) was assessed by measuring the width of the remaining wound area relative to the original wound width at 12 h post-treatment of EF-24 and TPA. Data represented the means ± SD from at least three independent experiments. ^a^ Significantly different, *p* < 0.05, when compared with the control group. ^b^ Significantly different, *p* < 0.05, when compared with the TPA treatment group. ^c^ Significantly different, *p* < 0.05, when compared with the TPA treatment plus EF-24 (0.25 μM) group.

**Figure 2:**
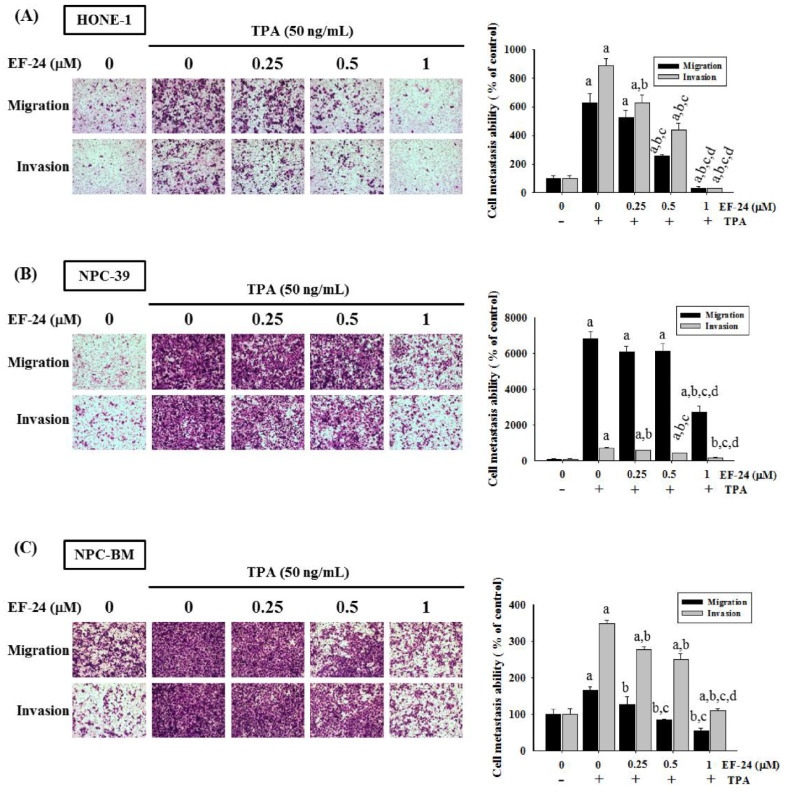
EF-24 impedes TPA-induced migrative and invasive responses in NPC cell lines. HONE-1 (**A**), NPC-39 (**B**), or NPC-BM cells (**C**) were incubated with various concentrations of EF-24 and TPA for 24 h. Cell migration and invasion were assessed in a Boyden chamber system coated without and with Matrigel, respectively. Quantification of responses is shown on the right. ^a^ Significantly different, *p* < 0.05, when compared with the control group. ^b^ Significantly different, *p* < 0.05, when compared with the TPA treatment group. ^c^ Significantly different, *p* < 0.05, when compared with the TPA treatment plus EF-24 (0.25 μM) group. ^d^ Significantly different, *p* < 0.05, when compared with the TPA treatment plus EF-24 (0.5 μM) group.

The inhibitory roles of EF-24 in cell invasiveness have been observed in numerous tumor cell lines [30,34,35]. Here, we first tested whether EF-24 affected the proliferation, motility, or invasion of NPC cells in response to TPA, a phorbol ester commonly used to promote cancer dissemination in cancer studies [12]. Our result demonstrated that there was no anti-proliferative effect of NF-24 on HONE-1, NPC-39, and NPC-BM cells, as only mild cytotoxicity was detected for HONE-1 cells at a high concentration (1 μM) (Figure 1B). Further examination of the cell motility by performing an in vitro wound healing revealed a dose-dependent inhibitory effect of EF-24 on TPA-induced NPC cell motility (Figure 1C–E). Moreover, TPA-induced NPC cell migration and invasion were consistently suppressed by various concentrations of EF-24 in HONE-1, NPC-39, and NPC-BM cells (Figure 2). These data implicate the usefulness of EF-24 in restraining NPC invasiveness and metastasis.

### 3.2. EF-24 Represses the Activity and Expression of MMP-9 in NPC

**Figure 3:**
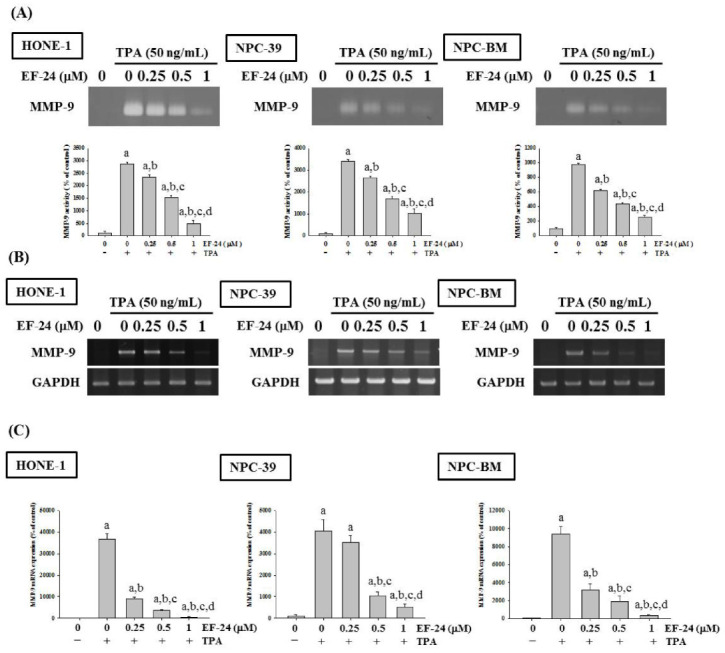
EF-24 suppresses the activity and expression of MMP-9 in NPC cells. HONE-1, NPC-39 and NPC-BM cells were pretreated with EF-24 for 1 h and incubated with TPA for an additional 23 h. Conditioned medium was collected for analyzing the activity of MMP-9 through gelatin zymography (**A**). Total RNA was extracted to assess the expression level of MMP-9 gene by using RT-PCR (**B**) and quantitative PCR (**C**). Densitometric analysis of gelatin zymography was conducted by the ImageJ software. The expression of the MMP-9 gene was normalized to the level of GAPDH gene. ^a^ Significantly different, *p* < 0.05, when compared with the control group. ^b^ Significantly different, *p* < 0.05, when compared with the TPA treatment group. ^c^ Significantly different, *p* < 0.05, when compared with the TPA treatment plus EF-24 (0.25 μM) group. ^d^ Significantly different, *p* < 0.05, when compared with the TPA treatment plus EF-24 (0.5 μM) group.

Since MMP-9 was shown to be a key player in mediating TPA-induced NPC cell invasion [47], we explored whether the levels of MMP-9 activity and expression were regulated during the inhibition of NPC invasion by EF-24. To test this, a gelatin zymography assay was used to evaluate the activity of MMP-9 in EF-24-treated NPC cell lines. We found that TPA-induced gelatin digestion in the culture media of all three NPC cell lines was decreased upon EF-24 treatment in a dose-dependent manner (Figure 3A), suggesting that EF-24 effectively represses the activity or extracellular abundance of MMP-9 in NPC. Furthermore, the intracellular expression levels of MMP-9 were also tested. It was demonstrated that EF-24 resulted in the downregulation of MMP-9 gene expression (Figure 3B,C) in all three NPC cell lines. These findings indicate that EF-24 reduced both the expression and the activity of a crucial determinant of NPC cell invasion.

### 3.3. EF-24 Negatively Regulates the Transcription of MMP-9 Gene by Interfering with Nuclear Translocation of NF-κB

**Figure 4:**
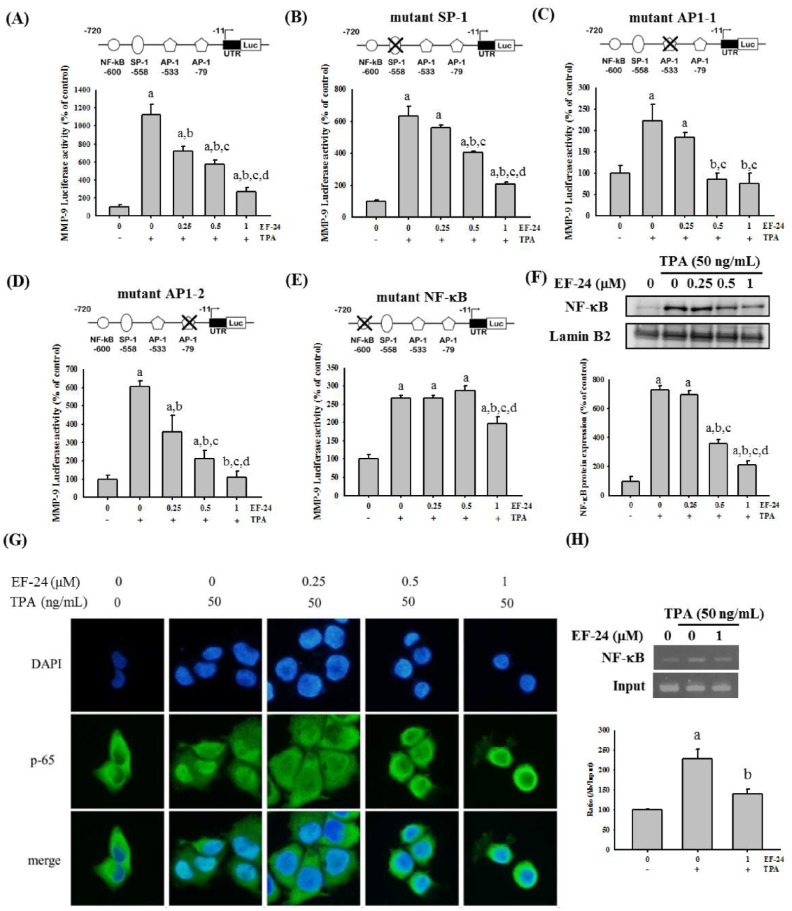
EF-24 suppresses MMP-9 gene transcription by interfering with nuclear translocation of NF-κB. HONE-1 cells expressing a luciferase gene driven by wild-type (**A**) and various mutated forms (**B**–**E**) of MMP-9 promoter were pretreated with EF-24 for 1 h and then incubated with TPA for an additional 23 h. Luciferase assays were used to measure the transcriptional activity of MMP-9 promoter. Schematic representations of the reporter plasmids containing a mutated SP-1 (**B**), AP-1 (**C**,**D**), or NF-κB responsive element (**E**) are shown on the top. To explore the nuclear translocation of NF-κB, a nuclear extract of untransfected HONE-1 cells after the treatment of EF-24 and TPA was isolated and analyzed for the protein levels of NF-κB (**F**). Blots are representative of three independent experiments. Densitometric analyses were conducted by ImageJ, and quantitative results were normalized to the nuclear levels of an internal control, lamin B2. In addition, HONE-1 cells treated with EF-24 and TPA were fixed and stained with DAPI and a specific antibody against NF-κB p65 (**G**). The interaction of NF-κB with MMP-9 promoter was analyzed by using chromatin immunoprecipitation assay (ChIP) with an NF-κB antibody in HONE-1 cells treated with or without NF-24 and TPA (**H**). Densitometric analyses were conducted by ImageJ. ^a^ Significantly different, *p* < 0.05, when compared with the control group. ^b^ Significantly different, *p* < 0.05, when compared with the TPA treatment group. ^c^ Significantly different, *p* < 0.05, when compared with the TPA treatment plus EF-24 (0.25 μM) group. ^d^ Significantly different, *p* < 0.05, when compared with the TPA treatment plus EF-24 (0.5 μM) group.

To further explore the mechanisms by which EF-24 affected MMP-9 gene expression, we performed a series of reporter gene assays by using various forms of mutated MMP-9 promoter. In HONE-2 cells expressing a reporter gene driven by wild-type MMP-9 promoter, EF-24 elicited a dose-dependent downregulation of the TPA-induced luciferase gene (Figure 4A), revealing a suppressive effect of EF-24 on MMP-9 gene transcription. While the SP-1 or AP-1 binding sequence of the MMP-9 promoter was mutated, EF-24 still contributed to a reduction in TPA-activated luciferase activities at a low (0.25 μM) or moderate (0.5 μM) concentration (Figure 4B–D). However, such reductions by a low or moderate concentration of EF-24 were restored as the NF-κB binding sequence was mutated (Figure 4E), suggesting that the regulation of MMP-9 gene transcription by EF-24 was mediated by the actions of NF-κB. Moreover, we tested whether EF-24 influenced the nuclear translocation of NF-κB. Our data from the isolated nuclear factions and immunofluorescence staining demonstrated a significant decrease in nuclear NF-κB levels under the treatment of EF-24 (0.5 and 1 μM) (Figure 4F–G). Uncropped blots are available in the supplementary material. Further chromatin immunoprecipitation experiments showed that EF-24 interfered with the TPA-induced interaction between the NF-κB and MMP-9 promoter (Figure 4H). Together, these findings indicate that EF-24 caused transcriptional suppression of MMP-9 in NPC cells by impeding the nuclear translocation of NF-κB.

### 3.4. EF-24 Inhibits JNK Signaling Pathway in NPC Invasion

**Figure 5:**
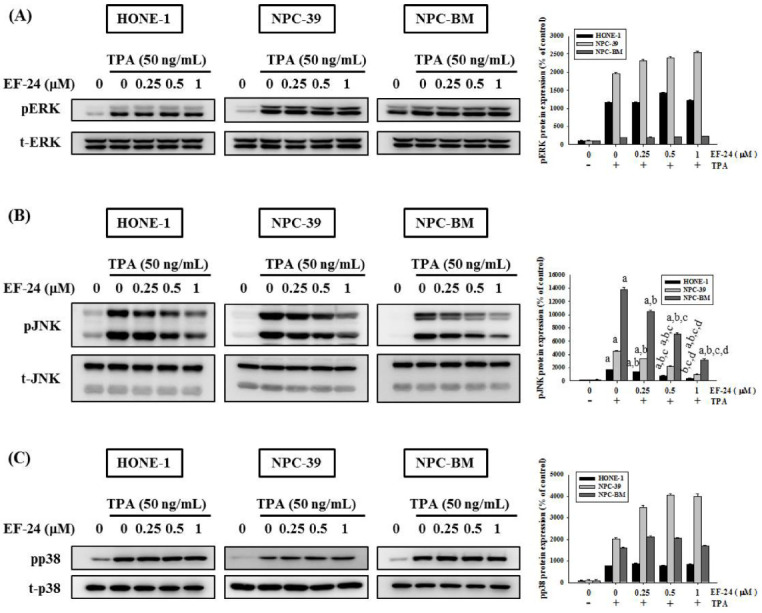
Regulation of EF-24 on MAPK activation. NPC cell lines were pretreated with EF-24 for 1 h and subsequently incubated with TPA for 23 h. Protein lysate was collected for a Western blot analysis to evaluate the phosphorylation of ERK (**A**), JNK (**B**), and p38 MAPK signaling (**C**). Densitometric data of kinase phosphorylation were obtained by ImageJ. Images shown are representative of three separate experiments. ^a^ Significantly different, *p* < 0.05, when compared with the control group. ^b^ Significantly different, *p* < 0.05, when compared with the TPA treatment group. ^c^ Significantly different, *p* < 0.05, when compared with the TPA treatment plus EF-24 (0.25 μM) group. ^d^ Significantly different, *p* < 0.05, when compared with the TPA treatment plus EF-24 (0.5 μM) group.

**Figure 6:**
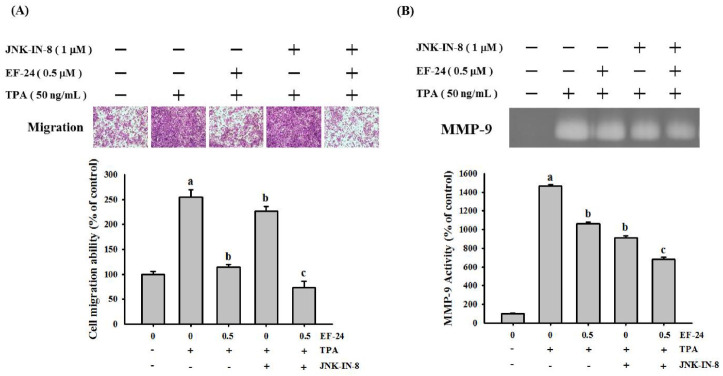
Synergistic effect of EF-24 and JNK-IN-8 on suppressing cell invasion and MMP-9 activity in NPC. HONE-1 cells were pretreated with EF-24, JNK-IN-8, or both for 1 h and then incubated with TPA for 23 h. Cell invasion responses were assessed by Boyden chamber assays (**A**), and MMP-9 activities were measured by gelatin zymography (**B**). ^a^ *p* < 0.05, compared with the untreated control; ^b^ *p* < 0.05, compared with TPA-treated cells; ^c^ *p* < 0.05, compared with the TPA + EF-24 treated cells.

It is known that many signaling pathways, including MAPK pathways [49], mediate MMP-9 expression and regulate cancer invasiveness. Subsequently, we explored whether EF-24 orchestrates the activation of ERK, JNK, and p38 in NPC cells. Our data showed that EF-24 inhibited the activation of JNK, whereas it failed to alter TPA-activated ERK and p38 in NPC cells (Figure 5). This observation is further supported by the finding that the use of EF-24 and a pharmaceutical inhibitor of JNK, JNK-IN-8 exhibited synergistic effects on suppressing TPA-induced invasion responses and MMP-9 activities in NPC (Figure 6).

## 4. Discussion

**Figure 7:**
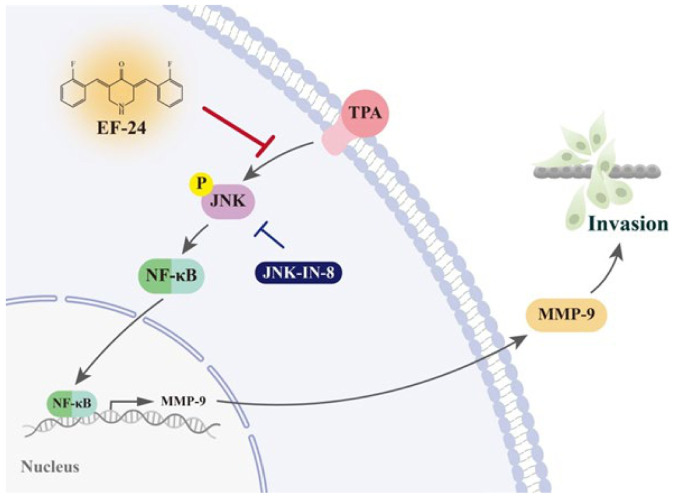
A schematic diagram of EF-24 in the regulation of TPA-induced NPC invasion.

Even though standard treatment for NPC has achieved prevailing outcomes in early-stage cases, metastatic cancer remains a substantial hurdle of NPC therapies. Therefore, extra options to treat this disease are necessary to enhance patients’ overall survival. A huge number of preclinical and clinical studies have revealed that curcumin, a herbal compound, increased the effectiveness of conventional cancer therapies, along with reducing their side effects and elevating the expression of anti-metastatic proteins, when given together with chemo- or radio-therapeutics [50]. In the present study, we showed that EF-24, a synthetic analog of curcumin with augmented bioavailability over its parent compound, rendered inhibitory effects on the invasiveness of NPC. Exploration of the underlying mechanisms demonstrated that the suppression of NPC invasion by EF-24 was coupled with a reduction in MMP-9 gene transcription by restraining the nuclear translocation of NF-κB and the activation of JNK signaling (Figure 7). Collectively, these results support the usefulness of EF-24 in controlling NPC progression.

Many investigations have demonstrated an inhibitory effect of curcumin on the proteolytic activities and expression levels of the gelatinases MMP-2 and -9 in a series of cancer cell lines [51,52,53,54]; such suppressions of various MMPs by curcumin were mainly caused by interference with the transcriptional activities of AP-1 and NF-κB [55]. Although EF-24 and its parent compound share numerous molecular mechanisms [56], such as the inactivation of NF-κB and the regulation of microRNAs, they likely exert bioactivities in different ways. For instance, curcumin blocked HIF-1α gene transcription, whereas EF-24 negatively regulated HIF-1α levels in a post-transcriptional manner [57]. In breast cancer cells, curcumin inhibited TPA-induced cell invasion and MMP-9 expression by suppressing both NF-κB and AP-1 activation [58]. However, our reporter assays showed that NF-κB, rather than AP-1, was functionally involved in the EF-24-mediated downregulation of MMP-9 in TPA-induced NPC cells. In addition to interfering with the activity of transcription factors, EF-24 was shown to resensitize renal cancer cells to TNF-related apoptosis-inducing ligand (TRAIL)-induced apoptosis via reducing intracellular ROS production and MMP-2/MMP-9 activity [37]. The findings from our and others’ studies implicate the use of EF-24 as a promising modality to restrain the anti-metastatic potential of nasopharyngeal cancers by targeting MMP-9.

MAPK pathways are known as a key determinant of cancer cell invasion that show a complex interplay with NF-κB signaling [59]. Unlike the highly consistent role of EF-24 in inhibiting NF-κB across different cancer types, the effect of EF-24 on regulating MAPK pathways is still under debate or appears to be specific to cancer/tissue types. In lung cancer cells, EF-24 induced cell apoptosis accompanied by the upregulation of three major MAPK pathways: ERK, JNK, and p38 [32]. On the contrary, EF-24 triggered oral cancer cell apoptosis through the deactivation of the MAPK/ERK signaling pathway [60]. However, we found that neither p38 nor ERK activation was affected by EF-24 in TPA-treated NPC cells. Notably, EF-24 inhibited JNK activation in TPA-treated NPC cells, and the co-treatment of NPC cells with EF-24 and a pharmaceutical inhibitor of JNK exhibited synergistic effects in suppressing TPA-induced invasion responses and MMP-9 activities.

Our data demonstrated an anti-cancer effect of EF-24 on NPC invasion. However, additional efforts are required to address some of the limitations of this study. One concern is that ingestion into the human body might influence the bioactivity of EF-24, although we observed a suppressive effect on the invasion responses of NPC cell cultures. Further in vivo investigations are needed to ascertain its clinical applications in the management of NPC. Another issue is that the cell lines tested here were Epstein–Barr virus (EBV)-negative. Nevertheless, the main histologic subtypes of NPC (nonkeratinizing carcinoma and undifferentiated carcinoma) are mostly positive for EBV infection [61]. Examining the efficacy of EF-24 on EBV-positive NPC cell lines will strengthen the clinical relevance.

## 5. Conclusions

In conclusion, our results showed that through interfering with the nuclear translocation of NF-κB and the activation of JNK, EF-24 inhibited MMP-9 gene transcription, repressing NPC invasion. These findings provide potential avenues for the use of curcumin analogs with increased bioavailability in managing this devastating disease.

## Data Availability

The data presented in this study are available upon request, from the corresponding author.

## Supplementary Materials

The following supporting information can be downloaded at https://www.mdpi.com/article/10.3390/cancers15051552/s1.
