# Supplementary material for: EF-24, a Curcumin Analog, Inhibits Cancer Cell Invasion in Human Nasopharyngeal Carcinoma through Transcriptional Suppression of Matrix Metalloproteinase-9 Gene Expression
Source: Cancers (Basel). 2023 Mar 1;15(5):1552. doi: 10.3390/cancers15051552 (PMC10000445; doi:10.3390/cancers15051552)
Supplement: Supplementary file 1 [file cancers-15-01552-s001.zip › cancers-2218038-supplementary.pdf]

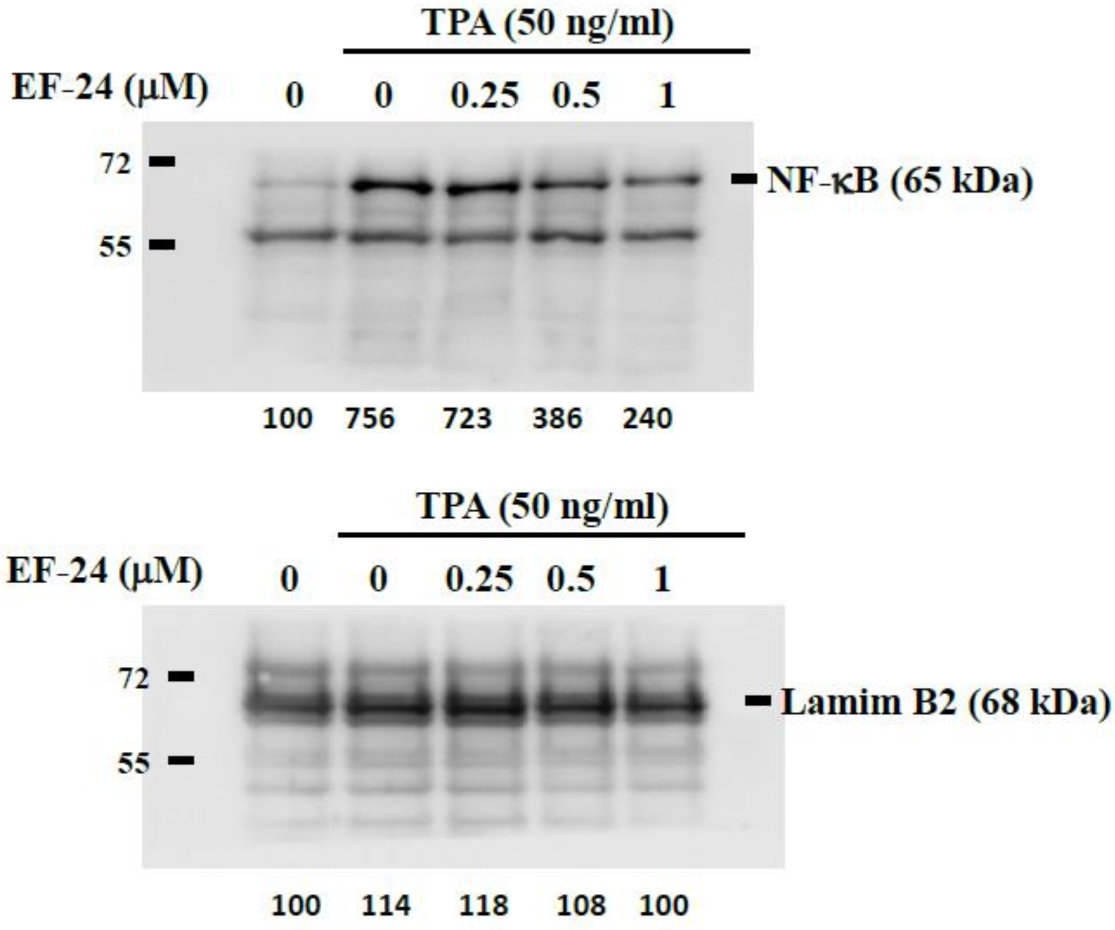

Figure S1. Uncropped western blot of figure 4F.

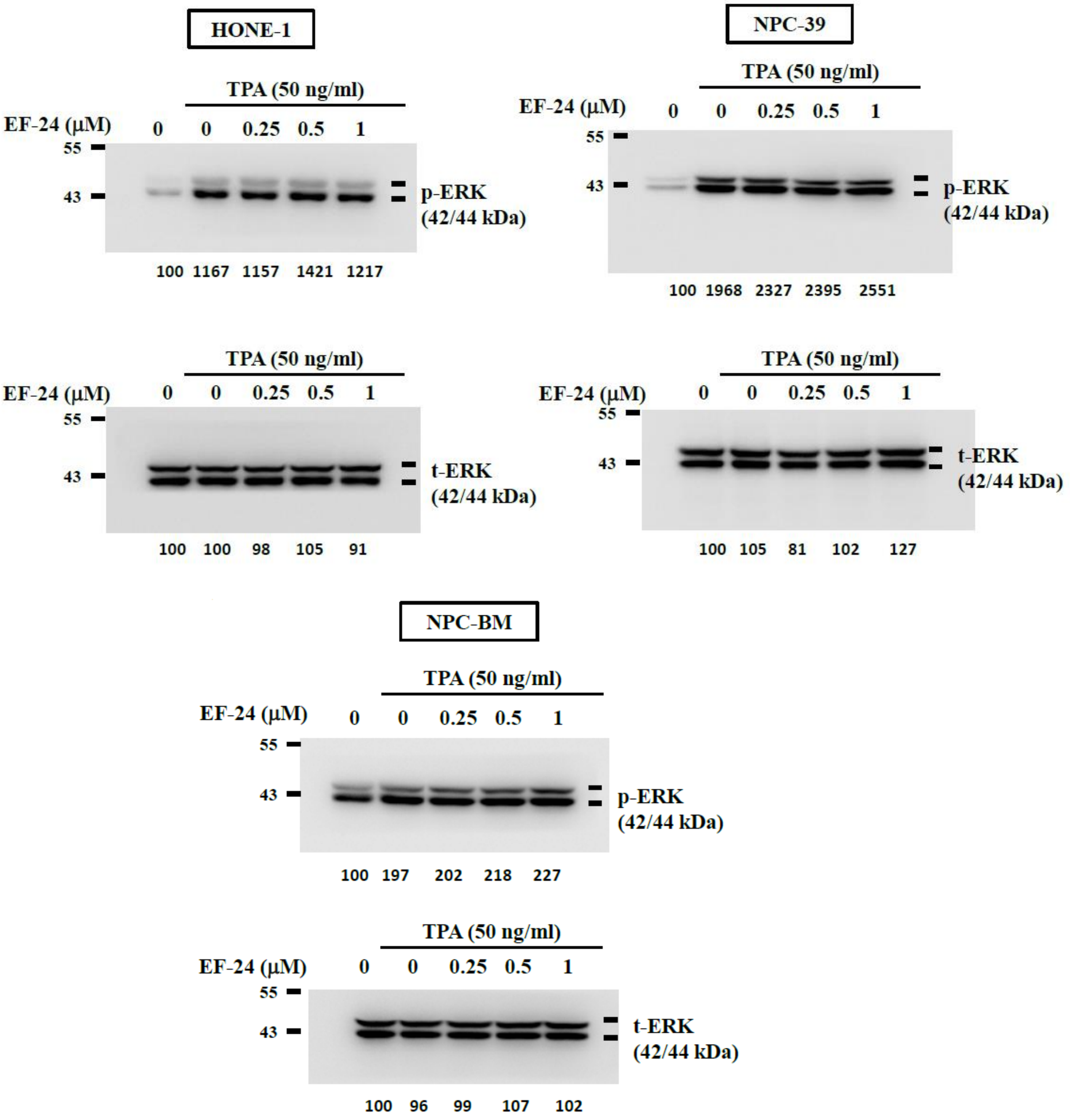

Figure S2. Uncropped western blot of figure 5A.

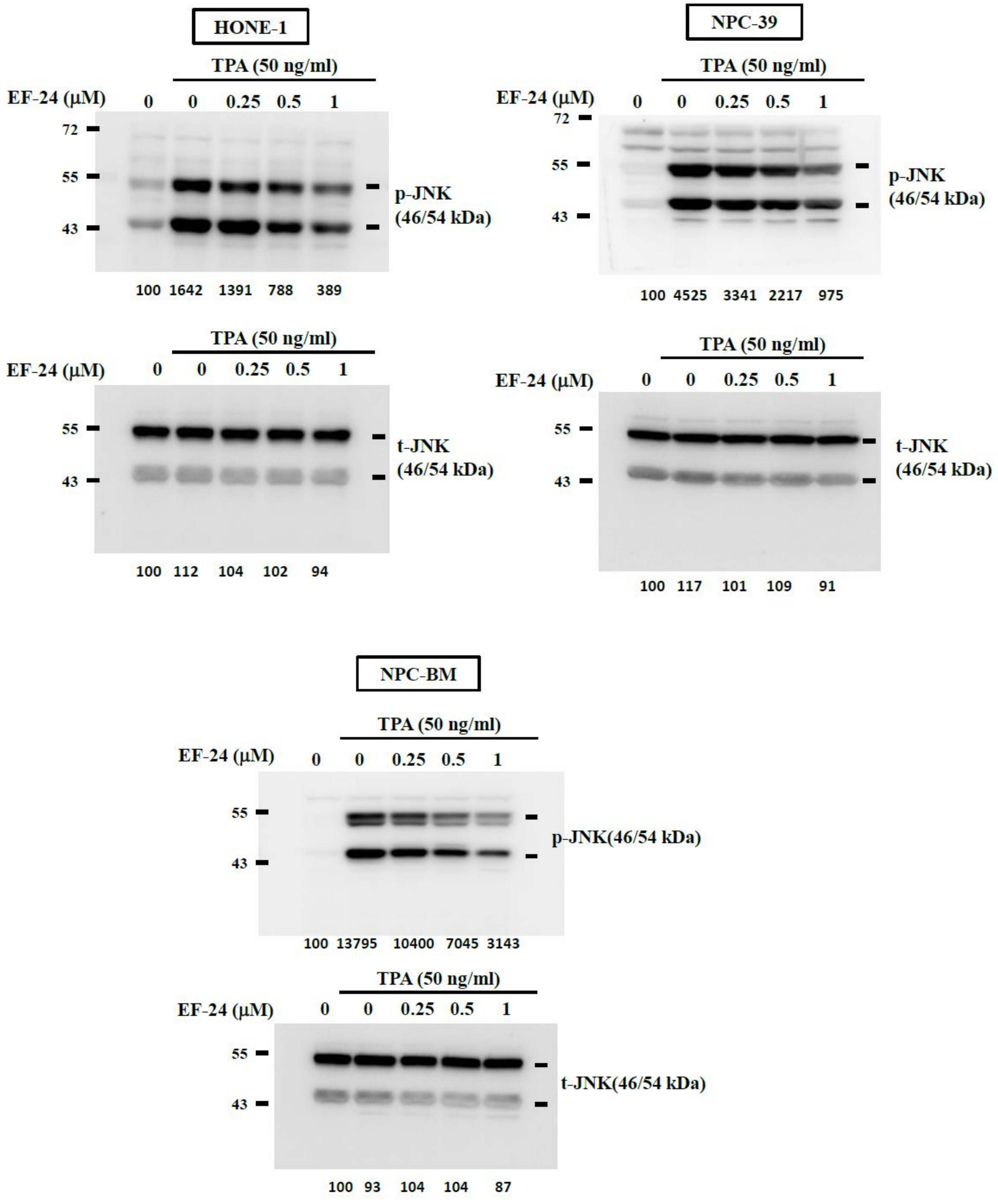

Figure S3. Uncropped western blot of figure 5B.

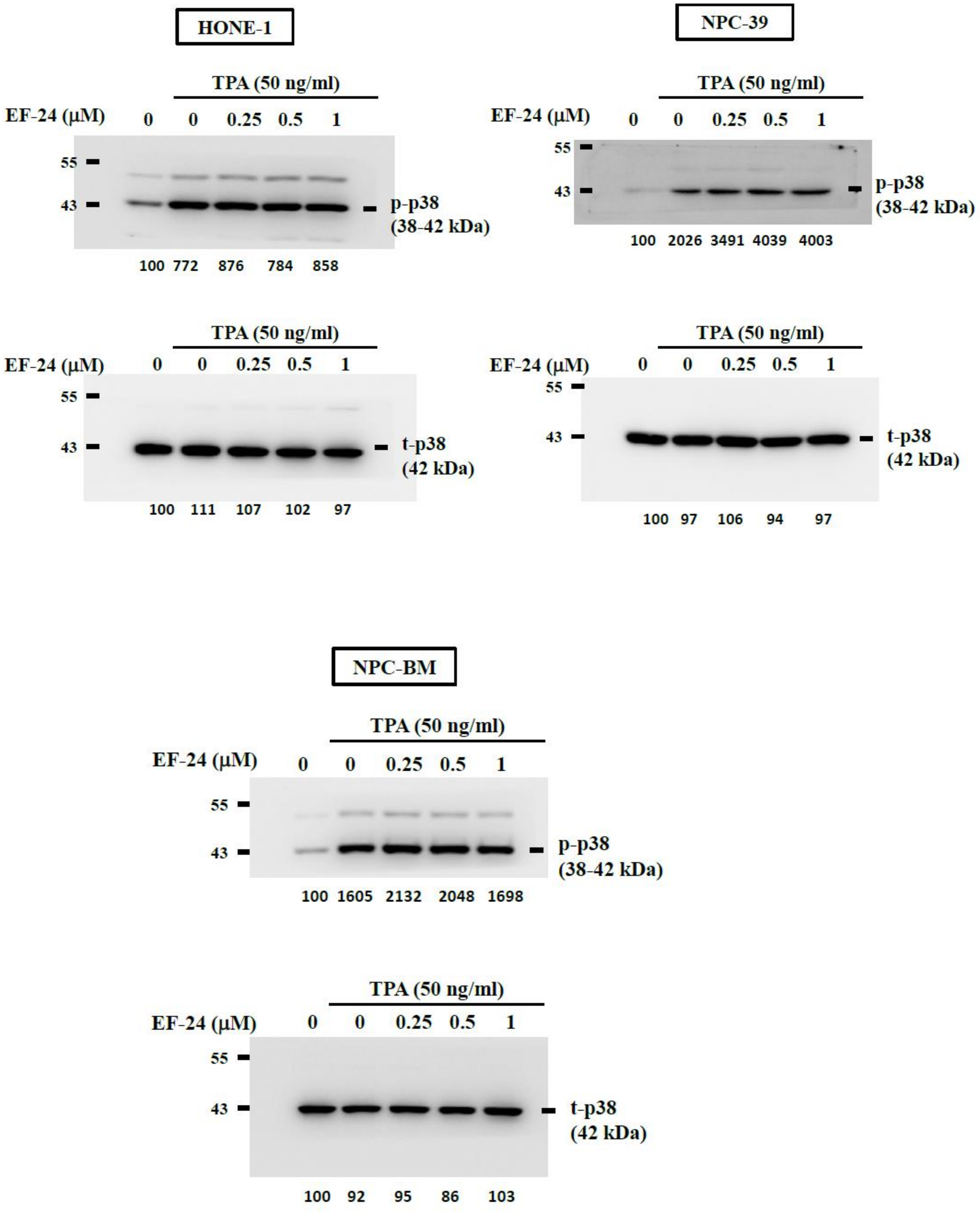

Figure S4. Uncropped western blot of figure 5C.
